# Cancer Mortality and Long-Term Environmental Exposure of Cadmium in Contaminated Community Based on a Third Retrospective Cause of Death Investigation of Residents Living in the Guangdong Province from 2004 to 2005

**DOI:** 10.1007/s12011-021-02599-0

**Published:** 2021-01-22

**Authors:** Aili Jiang, Lijuan Gong, Hao Ding, Mao Wang

**Affiliations:** 1 Department of Occupational and Environmental Health, School of Public Health, Sun Yat-sen University, 74 Zhongshan Road II, Guangzhou, 510080 People’s Republic of China; 2 ZhongShan Center for Disease Control and Prevention, Zhongshan, China; 3 Guangdong Technology Center of Work Safety Co., Ltd., Guangzhou, China

**Keywords:** Cancer mortality, Kriging, Environmental cadmium, Soil pollution

## Abstract

This study compared the temporal and geographic trends of cancer in China with a specific focus on the long-term exposure to soil cadmium (Cd) pollution. The geographic information system (GIS; kriging interpolation method) was used to detect the Cd contained in the soil from the Dabaoshan area, Guangdong Province. The standard rate ratio (SRR) was calculated to describe the relationship between Cd exposure and cancer mortality risk using the low-exposure group as a reference. Eight hundred six cancer deaths (533 male and 273 female) in the total population of 972,970 were identified, and the age-standardized rate (world) was 145.64 per 100,000. Significant dose-response relationships were found using the low-exposure group as the reference group. The Cd soil levels were positively associated with the cancer mortality risk in the community population, particularly for all cancers (SRR = 3.27; 95% CI = 2.42–4.55), esophageal cancer (SRR = 5.42; 95% CI = 1.07–30.56), stomach cancer (SRR = 5.99; 95% CI = 2.00–18.66), liver cancer (SRR = 4.45; 95% CI = 2.16–10.34), and lung cancer (SRR = 2.86; 95% CI = 1.62–5.31) for the total population. Additionally, similar results were obtained when using the 2000 China standard population. Cd exposure significantly affected the standardized mortality rates (China) by age group for all cancers, esophageal cancer, stomach cancer, liver cancer, and lung cancer in the total population, particularly in the age groups of 35–54, 55–74, and ≥ 75 years, respectively. Cd soil level is likely positively associated with increased cancer mortality of all cancer types and esophageal, stomach, liver, and lung cancers but not for other specific categories of cancer.

**Supplementary Information:**

The online version contains supplementary material available at 10.1007/s12011-021-02599-0.

## Introduction

Cadmium (Cd) is a chemical element that has been studied extensively. The Environmental Protection Agency (EPA) has classified Cd as a group B1 carcinogen (probable human carcinogen) based on sufficient evidence in animal studies but inadequate evidence from humans. Although occupational exposure to Cd could increase the risk of cancer, particularly lung cancer [1, 2], endometrial cancer [3], and pancreatic cancer [4], the association of environmental low-moderate level exposure to Cd and cancer is unclear. Moreover, most studies on Cd and cancer have focused on animals and occupational workers; however, few studies have determined and evaluated the cancer burden for community populations residing in Cd-contaminated areas for a long time.

Cancer has been the leading cause of death in China since 2010. For example, there were 3.12 million newly diagnosed cancer cases in 2012 according to the annual report of the Chinese Cancer Registry. During 1999–2008, the cancer incidence rates reached >2.4% per year, from 184.81 per 100,000 in 1989 to 286.69 per 100,000 in 2008 [5]. Recently, two national death surveys reported that the age-standard mortality rate of lung cancer and female breast cancer was increased by 33.25% and 32.89%, respectively [6]. Cancer can originate from environmental factors [7]. In 2013, Chinese authorities acknowledged the existence of so-called cancer villages [8], which could be due to pollution by harmful chemicals. In 2014, Shangba (Wengyuan County, Guangdong Province, PR China) was designated as a “cancer village” by nongovernmental organizations after it was reported by *Science* in 2014 that the area was heavily polluted by cadmium through the river and soil over approximately 4 decades [9].

Our previous study revealed probable associations between long-term environmental exposure to both cadmium and an increased risk of mortality from all cancers, as well as from stomach, esophageal, and lung cancers, only in three highly exposed villages (73,174 total population) from 2000 to 2007 [10]. However, little is known regarding the health effects and risk for the general residents residing in the far downstream and surrounding areas. Notably, rice, the most important food for China, can concentrate Cd from the environment. Several studies have used different populations to address this issue by calculating the intake of Cd from dietary intake [3, 11]. Dietary intake is only one of the pathways of Cd contamination from the environment, particularly in polluted soil [10].

A geographical information system (GIS) allows the investigation of the geographical distribution of environmental risk exposure. Chiang et al. conducted a GIS study on the relationship of Cd soil contamination and exposure of residents in Taiwan and found no association, suggesting that both the soils and residents are receptors of Cd as a pollutant from as yet unidentified sources [12].

Hence, we conducted a retrospective mortality study combining environmental exposure to address the association of nonoccupational Cd exposure and cancer mortality distribution in both Wengyuan County and Yingde City, Guangdong, China. The study was aimed at (1) identifying the potential Cd pollution area using soil samples through comprehensive modeling techniques (GIS Kriging), (2) measuring the exposure level of Cd in local soil samples by ICP-MS, and (3) investigating whether environmental exposure to cadmium is associated with an increased risk of cancer, particularly lung cancer.

## Methods

### Study Area

**Fig. 1:**
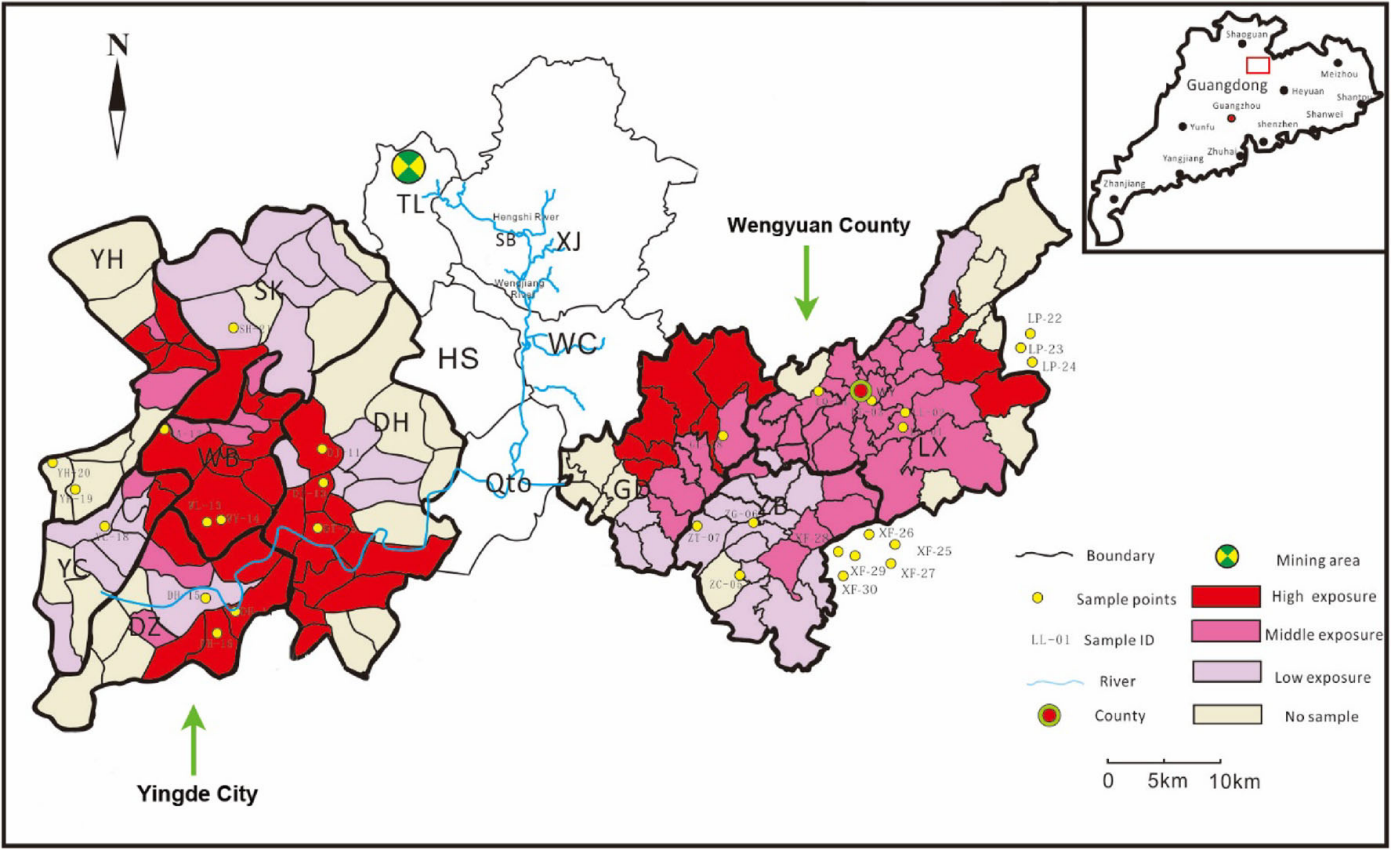
The cadmium exposure groups classified by cadmium concentrations of soil samples in each district in part of Wengyuan County and Yinde City (the colored area). Notes: Capital abbreviations represent villages and towns. Yingde City: YH, Yinghong; YC, Yingcheng; SK, Shakou; WB, Wangbu; DH, Donghua; DZ, Dazhan; HS, Hengshi; Qto, Qiaotou; Wengyuan County: GD, Guandu; ZB, Zhoubei; LX, Longxian; TL, Tielong; XJ, Xinjiang; WC, Wengcheng; blank area, no data on mortality

The Dabaoshan mine (24° 31′ 37″ N; 113° 42′ 49″ E) is located southeast of Shaoguan City between Wengyuan County and Qujiang County, Guangdong Province, China. The minerals found there include multimetal sulfide deposits, such as limonite in the superior part of the ore with a reservoir of approximately 20 million tons and copper sulfide lying in the inferior part with a reservoir of over 20 million tons [10]. The Hengshi River, as the main drainage pathway, has been heavily polluted by excretion water containing mine wastes and acid mine drainages (AMDs). This river delivers significant amounts of various heavy metals to numerous downstream villages [13]. The river runs through northwest Wengyuan County and southwest Yingde City and then flows in the Xijiang River. Wengyuan County is divided into one forestry center (Tielong) and seven towns (Xinjiang, Guandu, Zhoubei, Bazai, Jiangwei, Wengcheng, and Longxian), and Yingde City is divided into twenty-four towns. Each district and each administrative district boundary are shown in Fig. 1. Because cadmium exposure for soil acidification and the degree of pollution were different in each district, the population might have long-term exposure to cadmium at different levels. After nearly 40 years of exposure, some local residents in the Hengshi River area began to acquire enteron diseases, such as colon cancer; the area was reported to be a highly Cd-polluted area [9].

### GIS (Kriging Interpolation Method) and Exposure Assessment

To analyze the associations between the Cd exposure levels and cancer mortality, a three-level ordinal variable for Cd exposure was formed for statistical analysis by GIS.

ArcGIS 9.1 and its extensions were used to perform exploratory spatial data analysis and plot pollution prediction maps. We adopted a standardized geostatistical method [14] using ordinary kriging and indicator kriging models (TEPA threshold for food crop farmland, 5 mg/kg) to determine the range and distribution of Cd contamination in the study area. The Cd exposure levels for each county were from Cd measurements of soil samples that were within a certain distance to the centroid of the counties. If there was more than one sample within the specified distance from a county centroid, the mean of those samples was used as the exposure level for the county. It is unclear how far Cd contamination spreads in soil. Therefore, several distance bands were chosen: 5 km, 10 km, 15 km, and 20 km. The Cd exposure for each county was calculated under different distances separately. After Cd exposure assessment for each county, the Cd exposure was categorized as a high-risk area where the Cd exposure was higher than 70% of the counties, a median-risk area where the Cd exposure was lower than 70% and higher than 30% of the counties and a low-risk area where the Cd exposure was lower than 30% of the counties. According to the Cd concentration of the soil sampling in each village or community, this study divided the population residing in the region scope into three Cd exposure level groups by interquartile range (concentration ≤ 0.635 mg/kg was assigned as “low,” >0.635 mg/kg, and < 0.790 mg/kg were assigned as “medium,” and ≥ 0.790 mg/kg was assigned as “high”) in Fig. 1.

### Soil Sampling

The entire Wengyuan County and Yingde City regions were squared by latitude and longitude by 2 s on the map. We used the geographic information system to make a prediction map of the sampling points in the polluted area. There are totally 139 points marked in the map and 30 points selected according to the random sampling. There was 21 points located in the Wengyuan County and Yingde City, which was conducted a third retrospective cause of death investigation of residents living in the Guangdong province from 2004 to 2005 (Fig. 1). Meanwhile, there were three points located in the Lianping (LP) County and six points located in the Xinfeng (XF) County, which did not conduct a third retrospective cause of death investigation of residents living in the Guangdong Province from 2004 to 2005 (Fig. 1). Therefore, the soil samples from these 21 points were collected and measured in the laboratory in September 2013. The soil samples comprised at least five discrete samples taken in a 1 m × 1 m area using a trowel to sample surface soil (0–20 cm in depth) and were placed in plastic bags. The global positioning system (GPS) was used for precise positioning during the entire sample collection process.

### Sample Pre-treatment and Metal Analysis

In the laboratory, impurities were eliminated from the soil, and a 1-kg sample was placed in a polyethylene valve bag by quartering. After air-drying at room temperature, the soil samples were ground in an agate mortar and sieved with a nylon mesh (0.074 mm). All the sieved samples remained sealed with a polyethylene valve bag in the shade for analysis.

The metal elements in soil samples in this study, including cadmium, were detected in the Guangdong Province Key Laboratory of Geological Process and Mineral Resources Survey in School of Earth Science & Geological Engineering, Sun Yat-sen University. The inductively coupled plasma mass spectrometry (ICP-MS) instrument we used is Thermo Fisher X Serise-2 [15]. The key laboratory uses three standard products, two of which are reference materials from the US Geological Survey and National Certified Reference Materials of P. R. China, and the other mixed standard solution is produced by Inorganic Venture. Complete dissolution of the samples was performed using an acid digestion method (HNO_3_—HF) based on the procedure described by the State Key Laboratory of Geological Processes and Mineral Resources, China University of Geosciences (Wuhan).

### Study Population and the Collection of Cancer Case Mortality

Health effects were assessed by all-cancer and cancer cause-specific mortality. The third retrospective investigation of the cause of death from residents living in Guangdong Province in the period 2004–2005 was conducted in Guangdong Province, China, in August 2006 [16]. The protocol of the investigation was reported previously in detail [10]. Using a proportional allocation stratified cluster random sampling design, Yingde City and Wengyuan County were classed as rural areas I and II, respectively, according to the stratification methodology of the National Bureau of Statistics of China [16]. Agricultural farmers are the main population in the study area. Agriculture is the main source of economy in the study area, and agricultural products are self-sufficient and even exported. Therefore, they consume nearly all the products (e.g., vegetables, rice) produced on the local farm. Women generally did not have smoking or drinking habits in the Hengshi River region. All the reported deaths in the above-studied population had associated death certificates archived by local police departments, 89% of which were confirmed by a county physician. Moreover, all the death certificates were verified through door-to-door surveys or by well-trained investigators at local examination centers. To control for the quality of the analyses, strict measures were applied for every component of the survey, including the design stage, on-spot survey, data collection, sorting, and analysis. The coding used on the death certificates was previously validated by 12 trained oncologists. Because of a malignant neoplasm (codes C00-C97), the International Classification of Diseases, Tenth revision (ICD-10) was used to identify deaths. The outcomes were all-cause mortality and cause-specific mortality from major causes—that is, cancer (ICD-10 C00–97), cardiovascular disease (ICD-10 I00–99), coronary heart disease (ICD-10 I20–25), cerebrovascular disease (ICD-10 I60–69), and all other causes (ICD-10). The study was approved by the School of Public Health Ethics Committee, Sun Yat-sen University.

### Statistical Analysis

The average cancer mortality, such as the crude rate and age-standardized rate (ASR), of the three exposure groups was calculated using the Chinese standard population in 2000 and world standard population in 2000 as the standard population. The standardized rate ratios (SRRs) of the cancer mortality for the Cd exposure categories of medium and high were calculated by internal exposure estimation according to standard methods, using the mortality in the low-Cd-exposure category as the reference group. The SRR with a 95% confidence interval (CI), *χ*^2^ and *P* were calculated using the programs PEPI Compare2 and PEPT Describle for the Poisson statistical distribution [17].

## Results

### Cd Concentrations

Cd concentrations were found in 21 soil samples (mean = 0.691 mg/kg; median = 0.684 mg/kg; range = 0.335–1.516 mg/kg). The Cd concentration distribution in soil sampling was consistent with the historical data of Cd concentration in the nine previously studied villages [10]. The Cd soil levels were significantly higher than the government standard of 0.30 mg/kg (Soil Environmental Quality Standards, GB15618-1995).

### Cancer Mortality of Different Exposure Districts

806 cancer deaths (533 male and 273 female) in the total population of 972,970 were collected in the studied areas from 2004 to 2005. The age distribution was skewed: the median age was 65 years, and the 25th and 75th percentiles of the age distribution were 51 and 73 years old, respectively. The average ASR (China) and ASR (world) were 129.88 per 100,000 and 145.64 per 100,000, respectively. Table 1 shows the mortality of cancers by sex and exposure group. In the combined sex group and male and female groups separately, the ASR of the high-cadmium exposure group (HEG) was significantly higher than that of the medium-cadmium exposure group (MEG) and low-cadmium exposure group (LEG) (Table 1). Significant dose-response relationships were observed when using LEG as the reference group.

**Table 1:** Cancer mortality of three cadmium exposure groups by sex in study area from 2004 to 2005

Exposure group	Total population	Male				Female				Total			
		*N* value	Crude rate	ASR China	ASR world	*N* value	Crude rate	ASR China	ASR world	*N* value	Crude rate	ASR China	ASR world
Low	388,155	97	48.16	65.38	78.09	34	18.21	23.44	24.83	131	33.75	46.46	51.46
Medium	337,703	191	109.11	159.73	140.11	105	64.55	82.87	79.16	296	87.65	110.59	121.26
High	247,112	245	183.02	222.04	185.02	134	118.33	114.42	109.61	379	153.37	148.62	168.10
Total	972,970	533	104.45	168.91	198.95	273	59.01	88.42	92.33	806	82.84	129.88	145.64

*N*, the case of cancer patients

*ASR China*, age-standard rate by Chinese 2000 model population, per 100,000

*ASR world*, age-standard rate by world 2000 model population, per 100,000

### Standardized Rate Ratio for the Cancer Mortality of Major Cancer Sites with Exposure to Cd

We performed internal comparisons to examine dose-response relationships using the low-exposure group as the reference group (Table 2). The SRR (China) of all cancers in the MEG was 2.44 (95% CI = 1.85–3.30), and that of the HEG was as high as 3.40 (95% CI = 2.61–4.54) for male subjects; similar results were obtained for female subjects in all cancers. Most of the SRR values of the major cancer sites in the HEG were significantly higher than those in the LEG (SRR > 1.50). The data indicated strong associations between Cd exposure and stomach cancer (SRR_China_ = 6.24; 95% CI = 1.93–25.56), liver cancer (SRR_China_ = 4.19, 95% CI = 1.97–9.53), and lung cancer (SRR_China_ = 3.04; 95% CI = 1.58–5.68) for the total population between HEG and LEG, particularly regarding esophageal cancer (SRR_China_ = 4.56; 95% CI = 1.22–33.55). Additionally, similar results were obtained between the MEG and LEG. Thus, significant dose-response relationships were found for all cancers, stomach cancer, liver cancer, and lung cancer and male subjects in the esophagus.

**Table 2:** Mortalities and standardized rate ratios (SRR) of cancers for the medium and high-cadmium exposure groups by sex (by Chinese 2000 model population, per 100,000)

Cancer site	Male					Female					Total				
	L	M	H	SRR*	SRR^#^	L	M	H	SRR*	SRR^#^	L	M	H	SRR*	SRR^#^
C15	2.23	8.82	10.16	*3.96 (1.07–30.56)*	*4.56 (1.22–33.55)*	0.78	6.29	7.72	8.06 (0.73–276.20)	9.90 (0.90–315.51)	1.53	6.66	7.79	4.35 (0.67–34.53)	5.09 (0.92–27.52)
C16	2.36	22.82	33.49	*9.67 (3.17–72.35)*	*14.19 (4.68–102.19)*	3.39	10.84	9.88	*3.20 (1.09–16.37)*	2.91 (0.97–15.05)	2.86	15.40	17.85	*5.38 (1.57–21.62)*	*6.24 (1.93–25.56)*
C22	15.01	48.24	54.43	*3.21 (1.82–5.89)*	*3.63 (2.07–6.58)*	0.43	16.32	17.84	*37.95 (2.88–338.94)*	*41.49 (3.29–378.94)*	7.94	30.87	32.59	*3.89 (1.84–8.99)*	*4.19 (1.97–9.53)*
C33–34	22.57	38.18	54.68	1.69 (0.99–2.81)	2.42 (1.48–3.96)	1.91	17.32	30.27	*9.07 (2.02–75.86)*	*15.85 (4.22–93.24)*	12.55	23.79	38.19	1.90 (0.95–3.73)	*3.04 (1.58–5.68)*
C00–97	65.38	159.73	222.04	*2.44 (1.85–3.30)*	*3.40 (2.61–4.54)*	23.44	82.87	114.42	*3.54 (2.30–5.84)*	*4.88 (3.21–7.92)*	46.46	110.59	148.62	*2.38 (1.72–3.43)*	*3.20 (2.34–4.55)*

*C15* esophagus, *C16* stomach, *C22* liver, *C33–C34* trachea, bronchus, and lung, *C00–C97* all cancers

*L* low-cadmium exposure group mortality of age-standard rate (ASR) by Chinese 2000 model population, per 100,000; *M* medium-cadmium exposure group mortality of ASR by Chinese 2000 model population, per 100,000; *H* high-cadmium exposure group mortality of ASR by Chinese 2000 model population, per 100,000

SRR* SRR (95% CI) for medium-cadmium exposure group by using exposure group as the reference

*SRR*^*#*^ SRR (95% CI) for high-cadmium exposure group by using exposure group as the reference

—, not be calculated with none case

Italicized text, *P* value was significant at 0.05 level

High Cd exposure was positively associated with high cancer mortality risks in the community population, particularly for all cancers (SRR_world_ = 3.27; 95% CI = 2.42–4.55), esophageal cancer (SRR_world_ = 5.42; 95% CI = 1.07–30.56), stomach cancer (SRR_world_ = 5.99; 95% CI = 2.00–18.66), liver cancer (SRR_world_ = 4.45; 95% CI = 2.16–10.34), and lung cancer (SRR_world_ = 2.86; 95% CI = 1.62–5.31) in the total population (Table 3). Similar results were obtained for male and female subjects, excluding esophageal cancer (SRR_world_ = 3.47; 95% CI = 0.92–27.57) in male subjects and stomach cancer (SRR_world_ = 2.57; 95% CI = 0.70–8.41) in female subjects between the HEG and LEG.

**Table 3:** Mortalities and standardized rate ratios (SRR) of cancers for the medium and high-cadmium exposure groups by sex (by World 2000 model population, per 100,000)

Cancer site	Male					Female					Total				
	L	M	H	SRR*	SRR^#^	L	M	H	SRR*	SRR^#^	L	M	H	SRR*	SRR^#^
C15	2.35	7.19	8.16	3.06 (0.67–34.53)	3.47 (0.92–27.57)	0.95	6.09	7.41	6.41 (0.73–276.00)	7.80 (0.90–315.51)	1.65	7.56	8.95	4.58(0.80–38.67)	*5.42 (1.07–30.56)*
C16	3.57	20.80	25.78	*5.83 (1.92–17.88)*	*7.22 (2.44–21.82)*	3.67	9.66	9.42	2.63 (0.80–9.20)	2.57 (0.70–8.41)	3.62	16.83	21.69	*4.65 (1.51–14.72)*	*5.99 (2.00–18.66)*
C22	15.82	45.64	46.99	*2.88 (1.65–5.22)*	*2.97 (1.69–5.32)*	0.43	15.18	17.28	*35.30 (2.68–318.94)*	*40.19 (3.09–358.94)*	8.13	32.28	36.19	*3.97 (1.90–9.26)*	*4.45 (2.16–10.34)*
C33–34	27.52	30.52	47.12	1.11 (0.66–1.86)	*1.71 (1.05–2.71)*	2.02	16.65	28.68	*8.24 (2.26–54.44)*	*14.20 (4.07–90.25)*	14.77	27.75	42.27	*1.88 (1.00–3.58)*	*2.86 (1.62–5.31)*
C00–97	78.09	140.11	185.02	*1.79 (1.36–2.38)*	*2.37 (1.83–3.11)*	24.83	79.16	109.61	*3.19 (2.04–5.04)*	*4.41 (2.89–6.92)*	51.46	121.26	168.10	*2.36 (1.72–3.32)*	*3.27 (2.42–4.55)*

*C15* esophagus, *C16* stomach, *C22* liver, C33–C34 trachea, bronchus, and lung, C00–C97 all cancers

*L* low-cadmium exposure group mortality of age-standard rate (ASR) by Chinese 2000 model population, per 100,000

*M* medium-cadmium exposure group mortality of ASR by Chinese 2000 model population, per 100,000

*H* high-cadmium exposure group mortality of ASR by Chinese 2000 model population, per 100,000

SRR* SRR (95% CI) for medium-cadmium exposure group by using exposure group as the reference

*SRR*^#^ SRR (95% CI) for high-cadmium exposure group by using exposure group as the reference

—, not be calculated with none case

Italicized text, *P* value was significant at 0.05 level

### SRR for Cancer Mortality in Different Age Groups among the Three Cd Exposure Level Groups

**Fig. 2:**
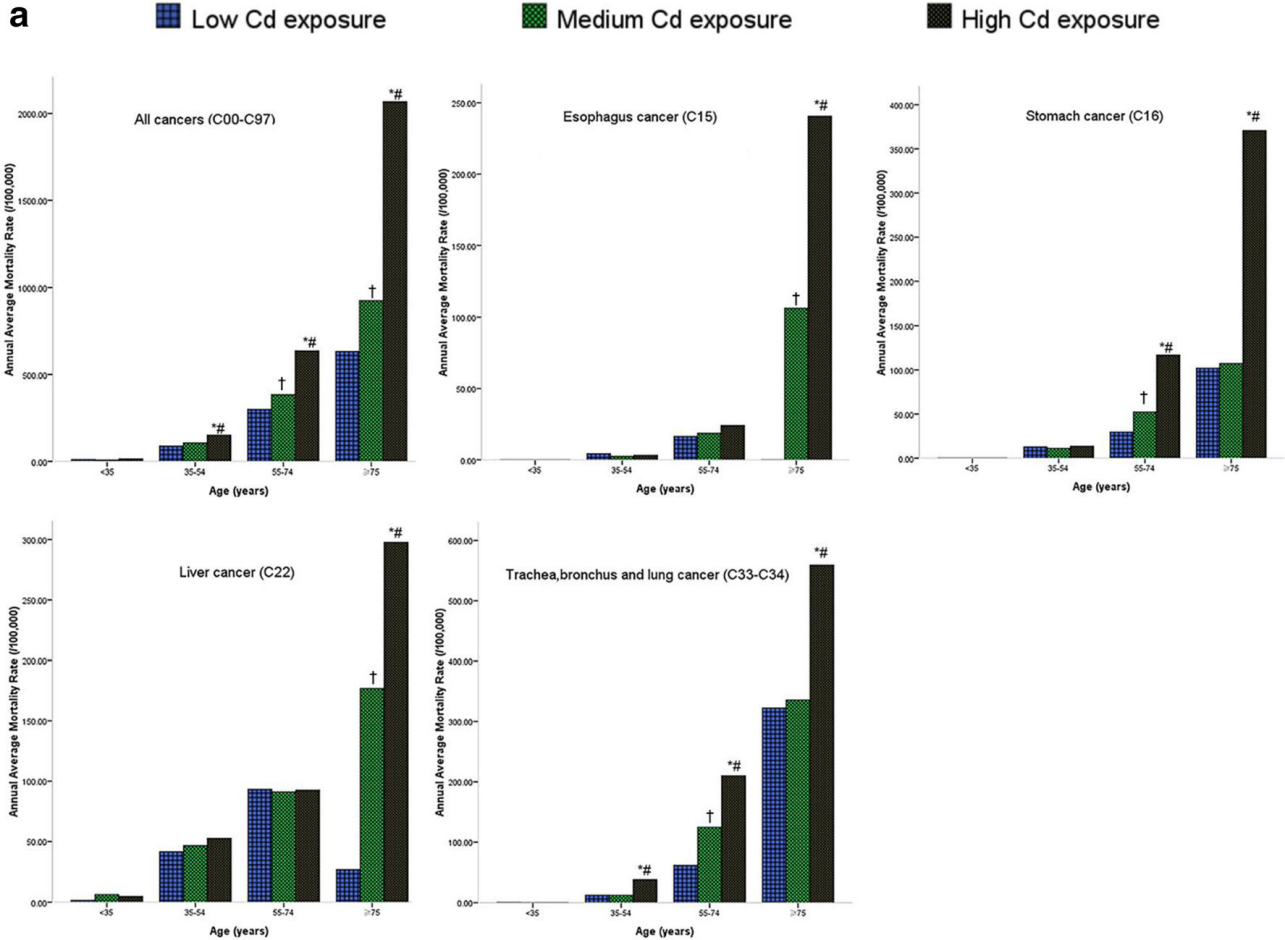

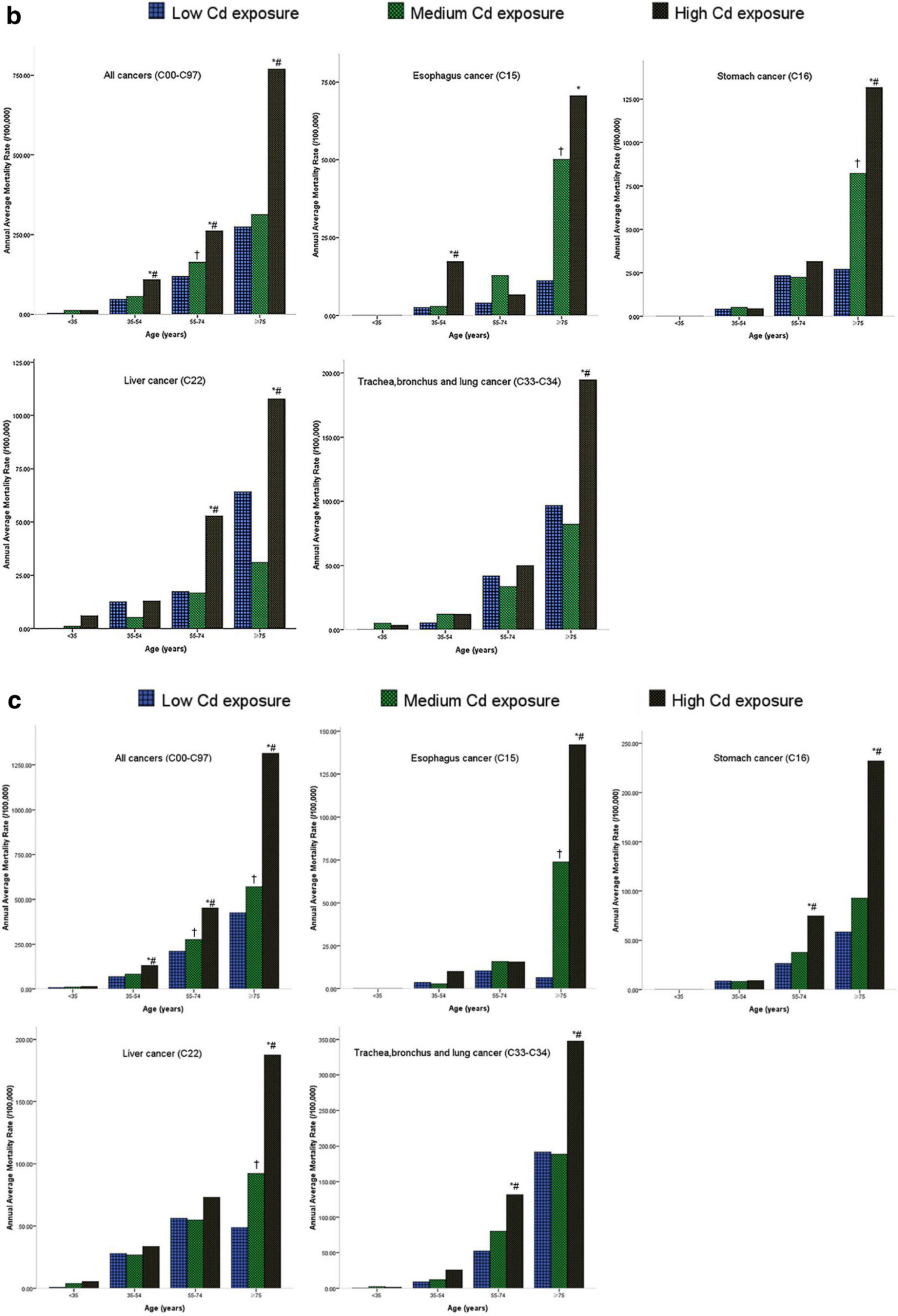
**a** Annual average mortality rate of all cancers, esophagus cancer, stomach cancer, liver cancer, and lung cancer by Cd exposure and age groups for male, using the 2000 China standard population. *The difference of the annual average mortality between high- and low-Cd exposure groups was statistically significant (*P* < 0.0167). ^#^The difference of the annual average mortality between high- and medium-Cd exposure groups was statistically significant (*P* < 0.0167). ^†^The difference of the annual average mortality between medium- and low-Cd exposure groups was statistically significant (*P* < 0.0167). **b** Annual average mortality rate of all cancers, esophagus cancer, stomach cancer, liver cancer, and lung cancer by Cd exposure and age groups for female, using the 2000 China standard population. *The difference of the annual average mortality between high- and low-Cd exposure groups was statistically significant (*P* < 0.0167). ^#^The difference of the annual average mortality between high and medium-Cd exposure groups was statistically significant (*P* < 0.0167). ^†^The difference of the annual average mortality between medium- and low-Cd exposure groups was statistically significant (*P* < 0.0167). **c** Annual average mortality rate of all cancers, esophagus cancer, stomach cancer, liver cancer, and lung cancer by Cd exposure and age groups for the total population, using the 2000 China standard population. *The difference of the annual average mortality between high- and low-Cd exposure groups was statistically significant (*P* < 0.0167). ^#^The difference of the annual average mortality between high- and medium-Cd exposure groups was statistically significant (*P* < 0.0167); ^†^The difference of the annual average mortality between medium- and low-Cd exposure groups was statistically significant (*P* < 0.0167)

Figure 2 shows significant differences in the annual average mortality rate (China) between the HEG and MEG, HEG and LEG, and MEG and LEG in different age groups, particularly in the 35–54-, 55–74-, and ≥ 75-year-old groups. Additionally, similar results were obtained when using the world standard population for subjects among the high-, medium-, and low-Cd exposure groups, as shown in Supplementary Fig. S1.

## Discussion

Heavy metal pollution, including lead, cadmium, arsenic, and mercury pollution, from sources such as mine exploitation, coal mines, coal-burning power plants, and agriculture using large amounts of fertilizer/pesticide, has become increasingly serious in China. Recently, concern has been growing about heavy pollution due to cadmium and cancer risks, as reported in 2014 [9]. Additionally, previous similar studies have mostly focused on occupational worker cadmium exposure, which might be limited by the healthy worker effect to draw conclusions [18]. In this study, we explored new evidence from general population data on the associations between cancer risk and long-term Cd exposure in the population by examining the concentration of Cd in neighboring soil samples. Our observations suggested that Cd exposure was associated with some cancers and differed from the occupational population. Chronic, lower-level, long-term residential oral exposure may be associated with a different clinical phenotype and spectrum of diseases in susceptible individuals, particularly in different aged-group populations.

The present study showed the distribution of Cd concentration with a median of 0.684 mg/kg in soil samples, far exceeding the government standard (Soil Environmental Quality Standards, GB15618-1995) of 0.30 mg/kg, which is a finding that was consistent with our previous studies. The Cd concentration in all 21 soil samples also exceeded the government standard. Previous studies have shown that local soil, water, and agricultural products such as rice have high levels of heavy metals (Cd) [19–23]. Sun et al. found that, in Guangdong, a southern China province, approximately 60% of soil samples exceeded the national soil Cd threshold, and each vegetable category, 4.6%, 29.4%, and 9.9% of the leafy vegetables, rootstalk vegetables, and fruit vegetables, respectively, exceeded the national limit for Cd in vegetables [24]. Most of the studied individuals were peasants, who ate rice as self-sufficient products in rural areas. Therefore, the distribution of Cd contamination and classification of Cd exposure levels were more reliable and precise [25].

In this study, the cancer cases were obtained from a third retrospective cause of death investigation of residents living in Guangdong Province from 2004 to 2005 conducted in August 2006. Therefore, the study population and causes of all deaths were complete and precisely validated. People residing in the Cd-contaminated areas were continuously and passively exposed to cadmium for nearly 40 years, and they were defined as three groups in the total population of 972,970 to explore the association between cancer risk and the Cd exposure level. The total population was nearly 13 times that in our previous studies, depending on the distribution and classification of Cd exposure [23]. Although the statistical power was restricted by the small number of cancer deaths, an increasing trend of cancer mortality was found from the LEG to HEG in all cancers, esophageal cancer, stomach cancer, liver cancer, and lung cancer, and the trend was more obvious in the older age group, in which cancer mortality was standardized by age and sex (Fig. 2). This finding may be explained by the cumulative Cd exposure levels increasing with age, and the inevitable organizational structure, and function changing with age.

The positive associations between Cd exposure and all types of cancer were consistent with the findings of other studies on environmental Cd exposure populations [26, 27]. At the community population level, our analysis showed that Cd exposure was significantly positively correlated with all-cancer mortality not only in the sexes combined but also in both males and female groups separately (Table 1). Moreover, the data revealed that, in the exposure group (35–54 years old), a significantly increasing trend of cancer mortality was found among low-Cd exposure, medium-cadmium exposure, and high-Cd exposure in all cancers. The age-standard mortality of the HEG was significantly decreased compared with that of the high-cadmium exposure area (HEA) in the previous study (Supplementary Table 1), with no difference compared with the rural areas of Guangdong Province (RAGDP) for female subjects and the total population [23]. This finding indicates that mortality represents all cancer-related mortality in the general population. However, the mortality of all cancers in the HEG group was significantly increased compared with that in the RAGDP group for male subjects, likely because the mortality of Cd exposure already affected male subjects. Nawrot et al. conducted a random population sample (*n* = 994) cohort with a 17-year follow-up and found that the overall cancer risk was significantly associated with a doubling of 24-h cadmium excretion (hazard ratio, 1.31; 95% CI, 1.03–1.65) in the high-exposure area [1]. For lung cancer, the adjusted hazard ratio was 1.70 (1.13–2.57; *P* = 0·011) for a doubling of 24-h urinary cadmium excretion, 4.17 (1.21–14.4; *P* = 0.024) for residence in the high-exposure area versus the low-exposure area, and 1.57 (1.11–2.24; *P* = 0.012) for a doubling of cadmium concentration in soil [1].

A strong positive association was found between environmental Cd exposure and lung cancer between both men and women. The results were consistent with our previous study and other studies. The age-standardized mortality of the HEG was not significantly different from that of the HEA in the previous study, showing no difference in the RAGDP for male subjects and the total population (Supplementary Table 1). This finding indicates that the mortality represents the lung cancer mortality of the general population. However, the mortality of lung cancer in the HEG was significantly increased compared with that in the RAGDP for female subjects, which may be explained by the mortality of Cd exposure affecting females. Our data showed that, in the exposure group (35–54 years), a significantly increased mortality was observed in the HEG compared with that in the MEG and LEG in men. In the exposure group (age ≥ 75 years), a significantly increased mortality was observed in the HEG compared with that in the LEG in women. The cause may be that male subjects were more affected than females, which probably included some confounding factors, such as smoking. Thus, for the 35-year-old population, exposure to Cd should be screened for lung cancer regularly.

The present study also showed that the mortality risk of esophageal cancer increased when the population was exposed to higher concentrations of Cd (Table 2), similar to our previous study finding. The age-standard esophageal cancer mortality of the HEG was significantly decreased compared with that of HEA in the previous study and was not different from that of the RAGDP among male, female, and total subjects. This finding indicates that the mortality represents the esophageal cancer mortality of the general population. Little evidence exists for an association between Cd exposure and esophageal cancer in the general population. The new data revealed that, among both male subjects and combined sexes in the exposure group (age ≥ 75 years), a significantly elevated mortality was observed in the HEG compared with that in the MEG and LEG. In the exposure group (age 35–54 and ≥ 75 years), a significantly elevated mortality was observed in the HEG compared with the LEG in female subjects. Compared with lung cancer, the cause may be that female subjects were more affected than male subjects, although some confounding factors, such as smoking and alcohol drinking, were excluded.

Regarding specific cancers, there is increasing concern about environmental Cd exposure and stomach cancer mortality. The age-standard stomach cancer mortality of HEG was significantly decreased compared with that of HEA in the previous study, with no difference compared with that of RAGDP (Supplementary Table 1). This finding indicates that mortality represents the stomach cancer mortality of the general population. In the present study, among both male subjects and the sexes combined in the exposure group (age 55–74 and ≥ 75 years), a significantly increased mortality was found in the HEG compared with that in the MEG and LEG, respectively; a significant elevation was found only in the exposure group aged ≥75 years in the HEG compared with that in the LEG in female subjects. This result suggests that the development of stomach cancer may be affected by Cd exposure in male subjects earlier than in female subjects. Although many risk factors for stomach cancer could not be controlled, such as *Helicobacter pylori* (*H. pylori*) infection of the stomach, smoking, alcohol, consumption of salt food, and a family history of stomach cancer, the population in the study area showed a similar socioeconomic status and dietary and geographic characteristics, which may limit the bias. Therefore, additional follow-up studies focusing on environmental Cd exposure are necessary to confirm these relationships.

Additional data supported the link of liver cancer mortality with Cd exposure (Table 2), which had not been found in other environmental and occupational studies. The age-standardized liver cancer mortality of the HEG was not significantly different from that of the HEA in the previous study in either male or female subjects, and not different from the RAGDP among male, female, and total subjects (Supplementary Table 1). This finding indicates that the mortality represents the liver cancer mortality of the general population. However, the liver cancer mortality in the HEG was significantly decreased compared with that in the HEA for the total population, likely because the level of Cd exposure was higher in the HEA than that in the HEG. The data showed that, among both male subjects and the sexes combined in the exposure group (age ≥ 75 years), a significantly elevated mortality was found in the high-Cd exposure groups compared with that in the medium- and low-Cd exposure groups. In the exposure group (age 55–74 and ≥ 75 years), a significantly elevated mortality was found in the HEG compared with that in the MEG and LEG among the female subjects. It is possible that Cd exposure affects the liver through pathways that involves drinking contaminated water and consuming contaminated food for a longer time than for lung cancer, possibly because Cd in the liver takes a longer time to accumulate. Similarly, some confounding factors could not be controlled in the present study, such as smoking, alcohol, aflatoxin levels, and hepatitis B virus infection, but further cohort studies are necessary to make a definitive conclusion.

Our study had the strengths of a large sample size, long-term exposure based on the community population, and adjustment for age and sex. Nevertheless, our study had some limitations. First, although the cancer mortality of the general residents living in the far downstream and surrounding areas was investigated, the lack of surface geography information—e.g., irrigation channels—could underestimate the Cd contamination exposure of the population. Second, potential confounders, such as smoking and occupation, were not adjusted in our study, which comprised areas with few industrial plants. Third, the interaction effect of Cd and other potential heavy metals on health outcomes was not addressed. Fourth, Cd exposure in other pathways could not be controlled in the study. Finally, the lack of indoor exposure measurements in the general population cannot accurately estimate health hazard risks.

The GIS link of environmental risk factors and clinical outcomes provides new opportunities for high-quality studies in a wide range of situations, creating a useful tool for administrators, planners, public health offices, and researchers. In future work, a geographically weighted regression model could be applied to both individual confounders and spatial factors at the same time. A larger sample size and longer follow-up must also be examined to determine the health risk in other populations. Our approach is much more convenient than traditional studies and provides a new method to screen and detect high-risk areas or populations. It can also be used to assess the cancer risk in high Cd-contaminated areas.

## Conclusion

This study revealed that the Cd soil levels might be positively associated with increased cancer mortality risk in community populations in different age groups. This finding suggests some significant links with an increased mortality risk of all cancers, lung cancer, esophageal cancer, stomach cancer, and liver cancer. Although further research is required to explore the effects of multiple heavy metals on cancer risk based on cohort studies, our study provides useful new insights into the causes of several types of cancer mortality and possibility of reducing these risks through environmental interventions.

## Supplementary Information

ESM 1 (DOCX 2105 kb)

## Data Availability

The datasets used or analyzed during the current study are available from the corresponding author on reasonable request.

## References

[1] Nawrot T, Plusquin M, Hogervorst J, Roels HA, Celis H, Thijs L, Vangronsveld J, Van Hecke E, Staessen JA (2006). Environmental exposure to cadmium and risk of cancer: a prospective population-based study. Lancet Oncol.

[2] Beveridge R, Pintos J, Parent ME, Asselin J, Siemiatycki J (2010). Lung cancer risk associated with occupational exposure to nickel, chromium VI, and cadmium in two population-based case-control studies in Montreal. Am J Ind Med.

[3] Akesson A, Julin B, Wolk A (2008). Long-term dietary cadmium intake and postmenopausal endometrial cancer incidence: a population-based prospective cohort study. Cancer Res.

[4] Luckett BG, Su LJ, Rood JC, Fontham ET (2012). Cadmium exposure and pancreatic cancer in South Louisiana. J Environ Public Health.

[5] Wanqing C, Zheng R, Hongmei Z (2012). The trend analysis of cancer mortality in China between 1989 and 2008. Chinese Journal of Oncolgy.

[6] Wanqing C, Zheng R, Siwei Z (2014). Cancer incidence, mortality and trend in China. Sience & technology review.

[7] Ahari SS, Agdam FB, Amani F, Yazdanbod A, Akhghari L (2013). Analysis of the relationships between esophageal cancer cases and climatic factors using a geographic information system (GIS): a case study of Ardabil province in Iran. Asian Pacific Journal of Cancer Prevention Apjcp.

[8] Mosbergen D (2013). China admits existence of ‘Cancer Villages’ in report, as pollution concerns mount, in the huffington post.

[9] Larson C (2014) Environmental science China gets serious about its pollutant-laden soil. Science 343 (6178):1415-1416. doi:DOI 10.1126/science.343.6178.141510 10.1126/science.343.6178.1415 24675928

[10] Wang M, Song H, Chen WQ, Lu C, Hu Q, Ren Z, Yang Y, Xu Y, Zhong A, Ling W (2011). Cancer mortality in a Chinese population surrounding a multi-metal sulphide mine in Guangdong province: an ecologic study. BMC Public Health.

[11] Sawada N, Iwasaki M, Inoue M, Takachi R, Sasazuki S, Yamaji T, Shimazu T, Endo Y, Tsugane S (2012). Long-term dietary cadmium intake and cancer incidence. Epidemiology.

[12] Chiang PH, Chan TC, Hsieh DP (2011). A GIS-aided assessment of the health hazards of cadmium in farm soils in central Taiwan. Int J Environ Res Public Health.

[13] Bao QS, Lu CY, Song H, Wang M, Ling W, Chen WQ, Deng XQ, Hao YT, Rao S (2009). Behavioural development of school-aged children who live around a multi-metal sulphide mine in Guangdong province, China: a cross-sectional study. BMC Public Health.

[14] Lin YP, Chang TK, Shih CW, Tseng CH (2002). Factorial and indicator kriging methods using a geographic information system to delineate spatial variation and pollution sources of soil heavy metals. Environ Geol.

[15] Xue-Qian WU, Qian-Sheng HU, Hong S, Nian L, Qing W, Mao W (2013) Analysis on the internal exposure levels of 14 elements in the long-term exposure groups in Dabao mountain,Guangdong. Chinese Journal of Health Laboratory Technology

[16] Ma WJ, Xu YJ, Zhang YR (2008). The research on death model and diseases burden in the residents of Guangdong province—the report on the 3 rd retrospective study on cause of death in Guangdong province, 2004–2005.

[17] Abramson JH (2005). WINPEPI (PEPI-for-Windows): computer programs for epidemiologists. Epidemiologic Perspectives & Innovations.

[18] Ju-Kun S, Yuan DB, Rao HF, Chen TF, Luan BS, Xu XM, Jiang FN, Zhong WD, Zhu JG (2016). Association between cd exposure and risk of prostate cancer: a PRISMA-compliant systematic review and meta-analysis. Medicine (Baltimore).

[19] Min ZJ, Zhi D, Yue S, Qiang LC (2004) Distribution and characteristics of heavy metals contaminations in soils from Dabaoshan Mine Area. Journal of Agro-environmental Science

[20] Yong-Gui WU, Chu-Xia L, Xiao-Li T, Wen-Zhou LU, Li-Xia Z, Cheng-Xing C, Ling Y (2005) Environmental impacts of acid mine drainage from the Dabaoshan Mine: I. Downstream aquatic ecosystem

[21] Wu YG, Lin C, Tong X, Lu W, Zhu L, Chu CX, Ling Y, Xu SJ (2005) Environmental impacts of acid mine drainage from the Dabaoshan Mine: II. Agricultural ecosystem

[22] Fu SM, Zhou YZ, Zhao YY, Zeng F, Gao QZ, Peng XZ, Dang Z, Zhang CB, Yang XQ, Yang ZJ, Dou L, Qiu RL, Ding J (2007). Study on heavy metals in soils contaminated by acid mine drainage from Dabaoshan mine, Guangdong. Huan Jing Ke Xue.

[23] Wang M, Xu Y, Pan S, Zhang J, Zhong A, Song H, Ling W (2011). Long-term heavy metal pollution and mortality in a Chinese population: an ecologic study. Biol Trace Elem Res.

[24] Sun FF, Wang FH, Wang X, He W, Wen D, Wang QF, Liu XX (2013). Soil threshold values of total and available cadmium for vegetable growing based on field data in Guangdong province, South China. J Sci Food Agric.

[25] Simmons RW, Pongsakul P, Saiyasitpanich D, Klinphoklap S (2005). Elevated levels of cadmium and zinc in paddy soils and elevated levels of cadmium in rice grain downstream of a zinc mineralized area in Thailand: implications for public health. Environ Geochem Health.

[26] Watanabe Y, Nogawa K, Nishijo M, Sakurai M, Ishizaki M, Morikawa Y, Kido T, Nakagawa H, Suwazono Y (2020). Relationship between cancer mortality and environmental cadmium exposure in the general Japanese population in cadmium non-polluted areas. Int J Hyg Envir Heal.

[27] Larsson SC, Alicja W (2016). Urinary cadmium and mortality from all causes, cancer and cardiovascular disease in the general population: systematic review and meta-analysis of cohort studies. Int J Epidemiol.

