# Supplementary material for: Cancer Mortality and Long-Term Environmental Exposure of Cadmium in Contaminated Community Based on a Third Retrospective Cause of Death Investigation of Residents Living in the Guangdong Province from 2004 to 2005
Source: Biol Trace Elem Res. 2021 Jan 22;199(12):4504–15. doi: 10.1007/s12011-021-02599-0 (PMC8516762; doi:10.1007/s12011-021-02599-0)
Supplement: Supplementary file 1 — (DOCX 2105 kb) [file 12011_2021_2599_MOESM1_ESM.docx]

**Cancer Mortality and Long-Term Environmental Exposure of Cadmium in contaminated community**

**Aili Jiang^1^, Lijuan Gong^1,2^, Hao Ding^3^,Mao Wang^1,*^**

^1^ Department of Occupational and Environmental Health, School of Public Health, Sun Yat-sen University, Guangzhou, China

^2^ ZhongShan Center for Disease Control and Prevention, Zhongshan, China

^3^ Guangdong Technology Center of Work Safety Co., Ltd. Guangzhou, China

* Corresponding author:

Mao Wang PhD, MD, Associate Prof

Department of Occupational and Environmental Health School of Public Health，Sun Yat-sen University 74 Zhongshan Road II Guangzhou 510080 PR-China

Tel 86-20-87335546

Fax 86-20-87330446

E-mail: [wangmao@mail.sysu.edu.cn](mailto:wangmao@mail.sysu.edu.cn)

**Journal: Biological Trace Element Research**


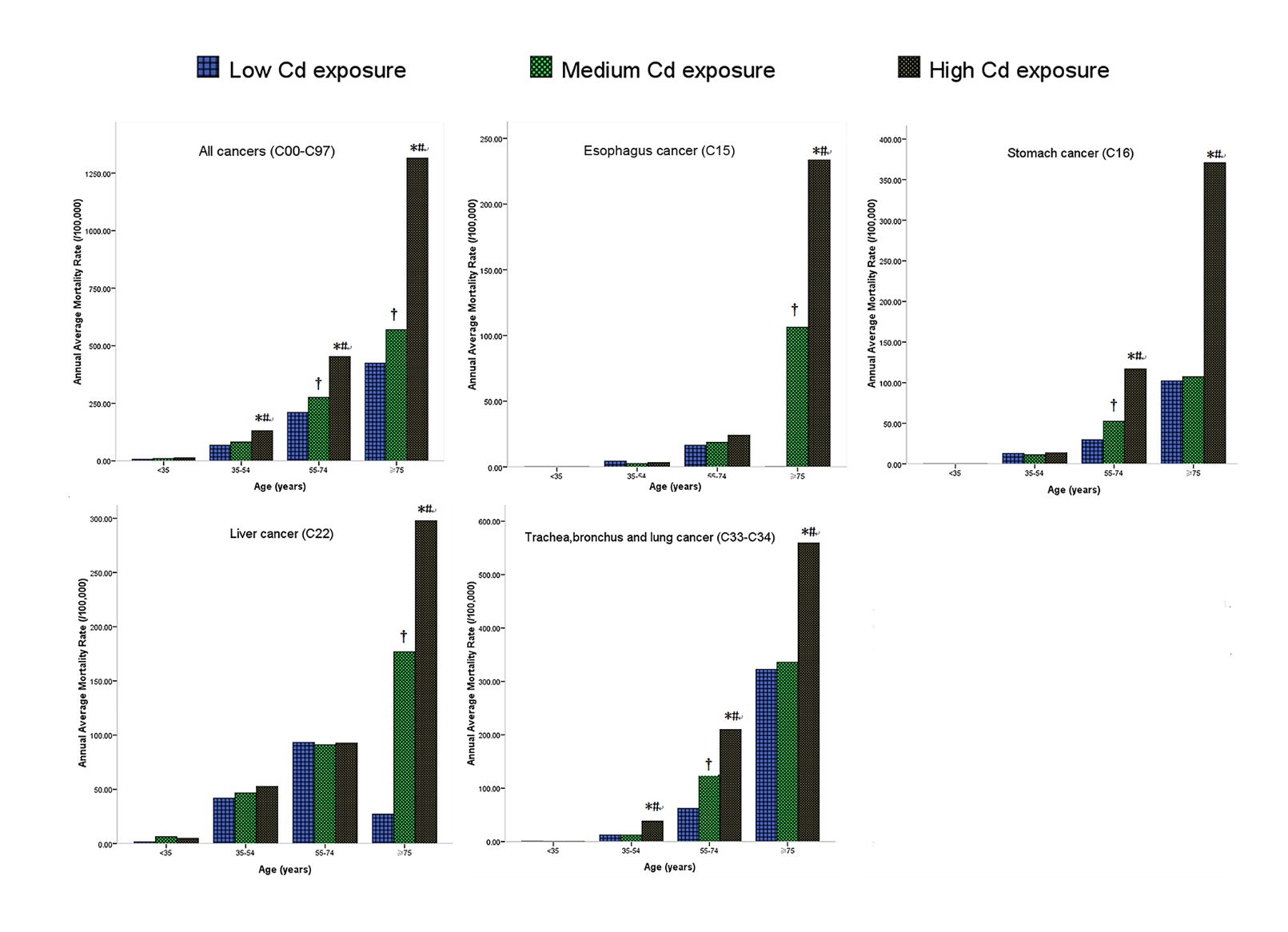


Fig. S1A. Annual average mortality rate of all cancers, esophagus cancer, stomach cancer, liver cancer and lung cancer by Cd exposure and age groups for male, using the 2000 world standard population.

^*^Indicating the difference of the annual average mortality between high and low-Cd exposure groups was statistically significant (*P*<0.0167);

^#^Indicating the difference of the annual average mortality between high and medium-Cd exposure groups was statistically significant (*P*<0.0167);

^†^Indicating the difference of the annual average mortality between medium and low-Cd exposure groups was statistically significant (*P*<0.0167).


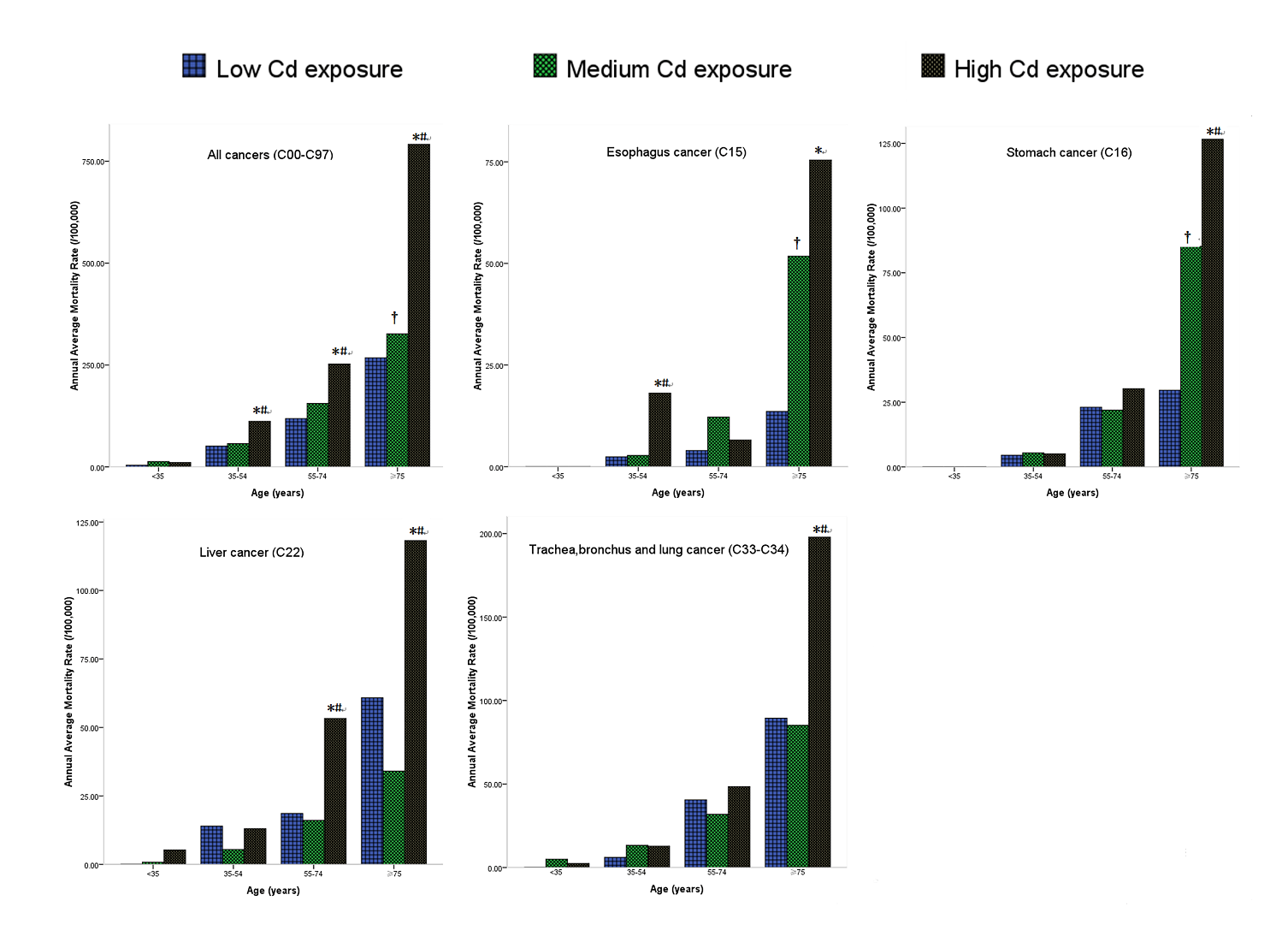


Fig. S1B. Annual average mortality rate of all cancers, esophagus cancer, stomach cancer, liver cancer and lung cancer by Cd exposure and age groups for female, using the 2000 world standard population.

^*^Indicating the difference of the annual average mortality between high and low-Cd exposure groups was statistically significant (*P*<0.0167);

^#^Indicating the difference of the annual average mortality between high and medium-Cd exposure groups was statistically significant (*P*<0.0167);

^†^Indicating the difference of the annual average mortality between medium and low-Cd exposure groups was statistically significant (*P*<0.0167).


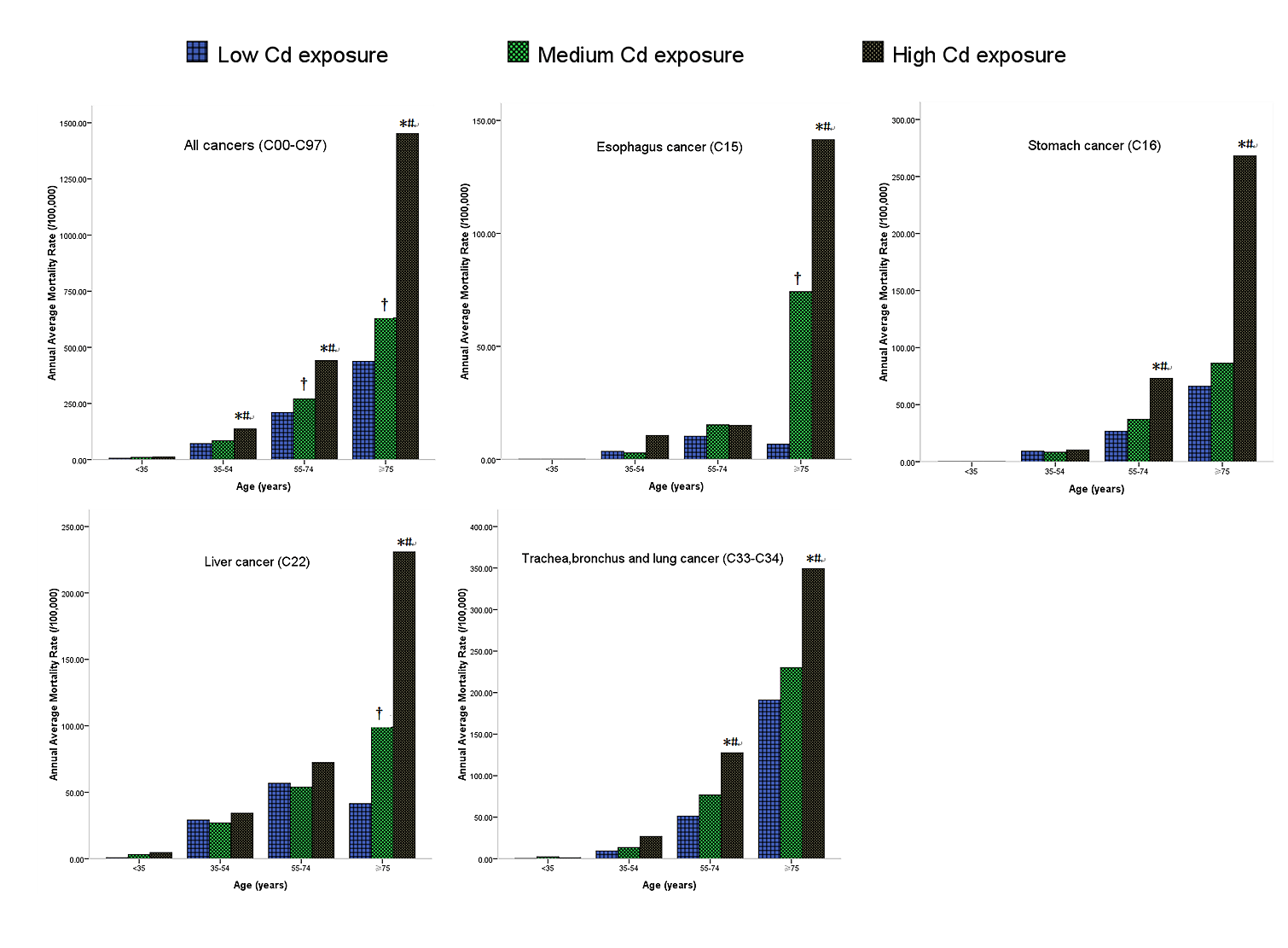
Fig. S1C. Annual average mortality rate of all cancers, esophagus cancer, stomach cancer, liver cancer and lung cancer by Cd exposure and age groups for the total population, using the 2000 world standard population.

^*^Indicating the difference of the annual average mortality between high and low-Cd exposure groups was statistically significant (*P*<0.0167);

^#^Indicating the difference of the annual average mortality between high and medium-Cd exposure groups was statistically significant (*P*<0.0167);

^†^Indicating the difference of the annual average mortality between medium and low-Cd exposure groups was statistically significant (*P*<0.0167).

**Supplementary Table 1** Comparison of the cancer mortality rates (per 100,000) in high-cadmium exposure groups (HEG) to the rates in high-exposure area (HEA) and the rural areas of Guangdong Province (RAGDP)

| Population | |  | |  | | | All cancers | | |  | Esophagus cancer | | | | |  | | Stomach cancer | | | | |  | | Liver cancer | | | |  | Lung cancer | | | | |  |  |  |
| --- | --- | --- | --- | --- | --- | --- | --- | --- | --- | --- | --- | --- | --- | --- | --- | --- | --- | --- | --- | --- | --- | --- | --- | --- | --- | --- | --- | --- | --- | --- | --- | --- | --- | --- | --- | --- | --- |
| Years | Statistic | | |  | Men | | | Women Total | | |  | | Men | Women Total | | |  | | Men | | Women Total | | |  | | Men Women Total | |  | | |  | Men Women Total | |  | |  |  |
| HEG  2004-2005 |  | | Rate^*^ | | | 222.0 | | | 114.4 148.6 | | | 10.2 | | | 7.7 7.8 | | | | | 33.5 | | 9.9 17.6 | | | | | 54.4 17.8 32.6 | | | |  | 54.7 | 30.3 38.2 | | | |  |
| Comparison:  HEA  2000-2007 |  | | Rate^#^  χ^2†^  P^†^ | | | 332.5  22.262  ＜0.001 | | | 191.1 263.8  19.469 32.088  ＜0.001 ＜0.001 | | | 45.2  22.279  ＜0.001 | | | 36.5 41.0  18.639 22.230  ＜0.001 ＜0.001 | | | | | 90.4  25.306  ＜0.001 | | 73.1 82.0  47.839 40.980  ＜0.001 ＜0.001 | | | | | 71.8 30.9 51.9  2.257 3.450 4.429  1.109 0.063 0.039 | | | |  | 63.9  0.681  0.409 | 33.7 49.2  0.250 1.391  0.617 0.238 | | | |  |
| Comparison: RAGDP  2004-2005 |  | | Rate ^*^  χ^2†^  P^†^ | | | 167.3  7.792  0.005 | | | 90.3 131.8  2.826 1.030  0.093 0.310 | | | 16.7  1.815  0.178 | | | 7.1 11.8  0.067 0.800  0.796 0.370 | | | | | 20.4  3.631  0.057 | | 9.9 15.0  0 0.273  1.000 0.601 | | | | | 63.1 16.7 39.8  0.693 0.029 0.671  0.405 0.866 0.413 | | | |  | 39.8  2.3700.121 | 14.6 49.6  5.001 1.862  0.025 0.172 | | | |  |
| ^*^ age-standard rate by Chinese 2000 model population, per 100,000;  ^#^ Rates were calculated using the expected deaths in the study regions divided by the total number of men and women as presented in **Reference 1**.  ^†^χ^2^ and P were calculated using the program PEPI Compare2. | | | | | | | | | | | | | | | | | | | | | | | | | | | | | | | | | | | | |  |
